# Supplementary material for: On-the-Fly Machine-Learned Force Fields for High-Fidelity Polymer Glass Transition Simulations
Source: J Chem Inf Model. 2026 Aug 28;66(17):11129–39. doi: 10.1021/acs.jcim.6c02116 (PMC13580126; doi:10.1021/acs.jcim.6c02116)
Supplement: Supplementary file 1 [file ci6c02116_si_001.pdf]

On-the-Fly Machine-Learned Force Fields for High-Fidelity Polymer Glass Transition Simulations

Ashutosh Srivastava, Sakshi Agarwal, Shivank Shukla, Harikrishna Sahu, and Rampi Ramprasad*

School of Materials Science and Engineering, Georgia Institute of Technology, 771 Ferst Drive, Atlanta, GA 30332

E-mail: ramprasad@gatech.edu

Table S1: Monomer units (M_{units}), polymer chains (N_{chains}), number of atoms (N_{atoms}), corresponding glass transition temperature (T_g), and coefficient of thermal expansion (CTE) of various PE structures.

$\mathbf{M}_{units}$	$\mathbf{N}_{chains}$	$\mathbf{N}_{atoms}$	T_g (K)	$\mathrm{CTE}_{Below T_g}$ (K^{-1})	$\mathrm{CTE}_{above T_g}$ (K^{-1})
33	1	206	233.0 ± 36.9	60.5×10^{-6}	574×10^{-6}
120	1	728	350.4 ± 70.2	60.7×10^{-6}	543.3×10^{-6}
33	8	1648	303.6 ± 49.6	82×10^{-6}	513×10^{-6}
33	27	5562	315.0 ± 27.2	80.6×10^{-6}	523.7×10^{-6}

Glass Transition Temperature of Polyethylene Obtained from Independent Replica and Cooling-Rate Simulations

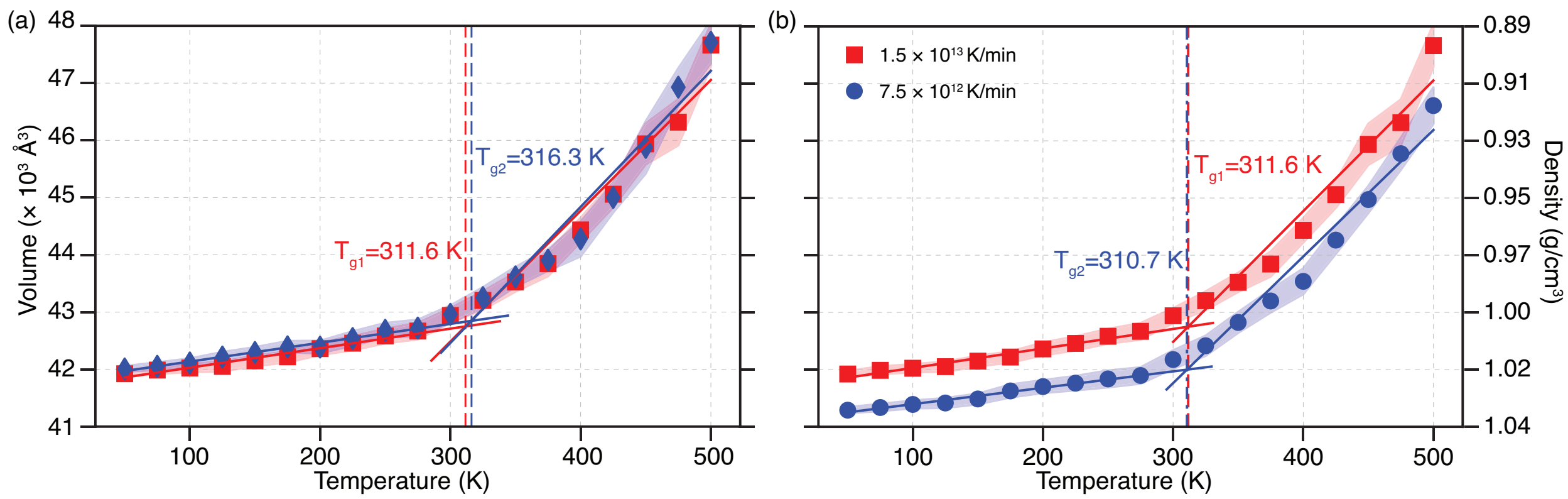

Figure S1: Polyethylene glass transition temperature (T_g) obtained from (a) replica runs and (b) two cooling-rate simulations. Shaded regions denote the standard deviations in volume/density at each temperature. Solid lines show the fitted curves used to extract T_g, and dashed lines indicate the corresponding T_g values.

Weighted Flory–Fox Extrapolation of T_g to Infinite Molecular Weight

The dependence of T_g on molar mass was analyzed using the Fox-Flory relationship by plotting T_g as a function of inverse molar mass ($1/M$). A weighted linear regression was performed, with the weighting factors set to the inverse of the calculated OTF-MLFF uncertainties to account for measurement precision. The resulting fit was extrapolated to $1/M = 0$, corresponding to the infinite molar mass limit ($M \to \infty$). The intercept of the weighted regression line represents the estimated glass transition temperature at infinite molecular weight, T_g^∞, while the associated uncertainty was obtained from the covariance matrix of the fit. Experimental data are shown with their respective error bars, the weighted linear fit is indicated by the dashed line, and the extrapolated T_g^∞ value is highlighted at $1/M = 0$ with its confidence interval. This approach provides an estimate of the asymptotic glass transition

temperature and evaluates the applicability of the Flory–Fox model to the molecular weight dependence.

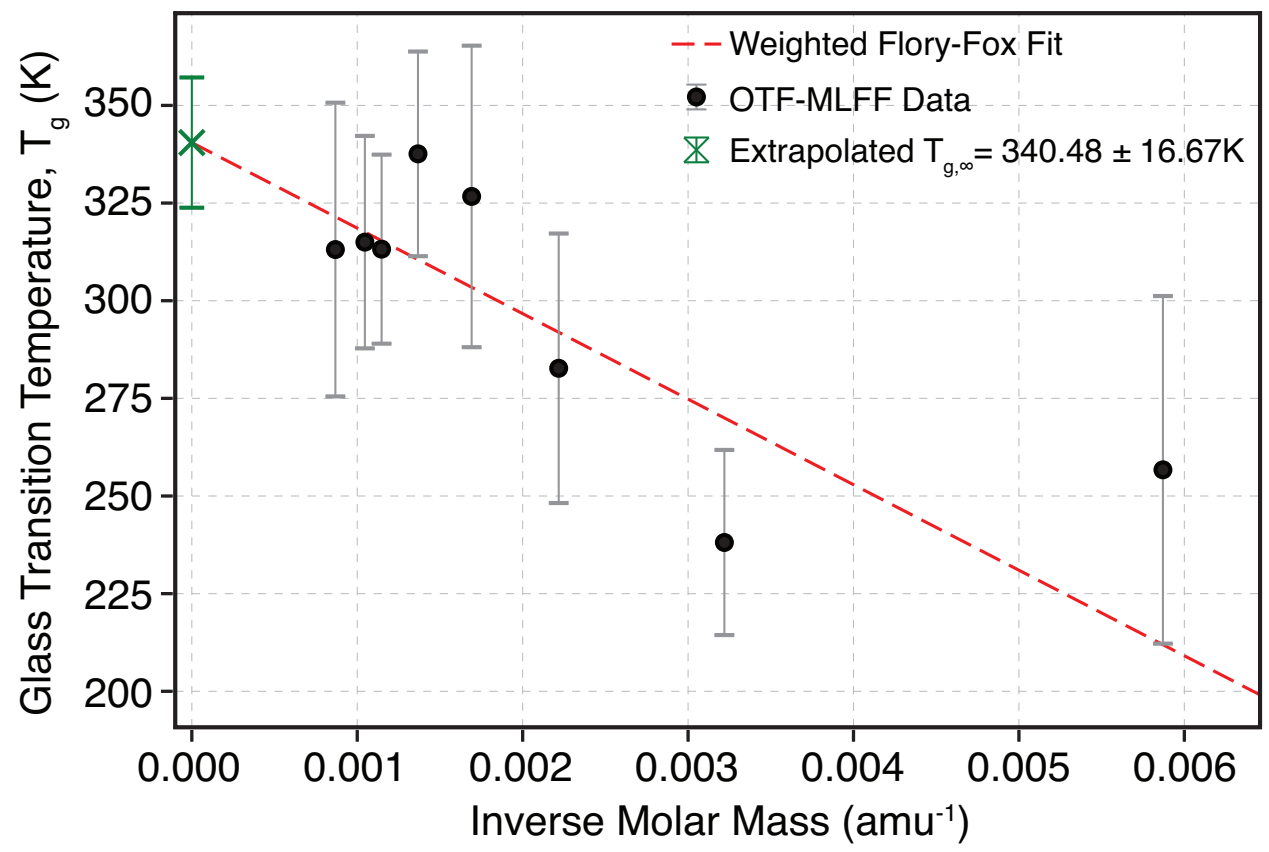

Figure S2: Weighted Flory–Fox extrapolation of glass transition temperature (T_g) as a function of inverse molar mass ($1/M$), showing the determination of the infinite-molecular-weight glass transition temperature, T_g^{∞}

Step-by-step MLFF Generation Protocol and Extracting Glass Transition Temperature

1. **Initial polymer structure generation:** The initial structure of each polymer was generated using Polymer Structure Predictor (PSP). The number of monomers for each polymer was selected in such a way that the total number of atoms in an amorphous polymer structure is around 200-250 atoms. This is essential, keeping in mind that the configuration sampling will be performed using AIMD.

2. **AIMD OTF sampling and preparation of MLFF:** Keeping the total number of atoms around 200-250 assists in performing AIMD with on-the-fly (OTF) training and sampling (implemented in VASP) for a sufficiently large time scale of 200 ps, which was not possible earlier with pure AIMD. The OTF learning approach is a hybrid approach of first principles and machine learning. First principles calculations were only executed when a new configuration was encountered for which the Bayesian

error exceeded the threshold and which was not sampled earlier during the ab-initio molecular dynamics (AIMD) run. Similarly, for configurations very close to those already sampled, the approach uses a machine learning algorithm to predict first-principles output parameters, such as forces, energies, and stresses. In this way, we collect all distinct configurations along with their atomic positions and forces, which are then used to build the machine learning force field. OTF-AIMD simulations have been performed using an isothermal–isobaric (NPT) ensemble, allowing the cell shape and volume to relax during dynamics.

3. **Building a larger polymer structure having multiple chains (as used in training):** The equilibrated structures at the respective temperatures were then used to build a larger polymer structure having multiple such repeat units. This was validated by testing the approach with longer chain lengths and multiple repeat-unit systems of polyethylene (see the "testing scalability across system sizes" section of the manuscript). We repeated the chains to build the larger polymer structure (containing around 5000-8000 atoms).

4. **Performing T_g simulations:** Starting from high temperatures (600-800K), structures were cooled to 50K using a 25 K temperature ramp. At each temperature, structures were equilibrated for 100 ps. We have extracted average volume/density information from the last 50ps production run.

5. **Selection of points for linear-fit lines:** The selection of points for the linear fits in the glassy and the rubbery regimes was performed using an automated procedure rather than manual selection. Specifically, the temperature-volume data were partitioned into two linear regimes by systematically testing the possible splits between 25% and 75% of the dataset, while requiring at least three points in each regime. For each candidate split index (i), an independent linear regression was performed on the low and high-temperature segments. The quality of each fit was quantified using the coefficient

of determination (R^2), and a weighted combined metric was computed as:

$$R^2_{combined} = \frac{R_1^2 N_1 + R_2^2 N_2}{N_1 + N_2} \tag{S1}$$

here R_1^2 and R_2^2 correspond to the two linear fits, and N_1, N_2 are the number of points in each segment. The optimal split point was selected as the one maximizing the $R^2_{combined}$. The intersection of the resulting linear fits was then used as the estimate for the glass transition temperature. Therefore, removing the subjective bias in point selection provides a reproducible criterion for identifying the glassy and rubbery regimes.

6. **Error Propagation and 66.67% Confidence Interval Estimation for Intersection Analysis** To estimate the uncertainty associated with the glass transition temperature, T_g (intersection point of two linear regression lines) was evaluated using the analytical error propagation method based on first-order Taylor expansion. The intersection point $T_g = \frac{b_1 - b_2}{m_1 - m_2}$ and corresponding volume, $V = m_1 T_g + b_1$ were determined from the regression slopes (m_1, m_2) and intercepts (b_1, b_2) obtained from least-square fitting. Then the propagated uncertainties were calculated using the following relation,

$$(\delta f)^2 = \sum_i (\frac{\delta f}{\delta x_i})^2 (\delta x_i)^2 \tag{S2}$$

Here, δx_i are the standard errors associated with the variables. The intercept uncertainty were then calculated using the following relation;

$$\sigma_b = \sigma_m \sqrt{\Sigma x^2 / n} \tag{S3}$$

where σ_b and σ_m are the standard errors for the intercepts and slopes, respectively. The partial derivative of T_g were calculated with respect to m_1, m_2, b_1, and b_2 to account for the correlated error contributions from both the regressions. The final uncertainty in determining T_g was obtained as the square root of the summed variance contributions.

The 95% confidence interval was then calculated as,

$$CI_{66.67\%} = T_g \pm 0.97\sigma_{T_g}, \tag{S4}$$

where σ_{T_g} is the standard error derived from the above propagation formula. This approach provides a good quantification of the uncertainty in the T_g values.

Table S2: Details of monomer units (M_{units}), number of atoms in the training unit cell (N_{atoms}), Number of configurations sampled during MLFF training ($N_{sampled}$), training temperatures (T_{train}), and simulated supercell (($N_{atoms})_{ss}$) used for T_g calculations of the polymers studied in this work.

**SN**	**Polymer smiles**	$\mathbf{M}_{units}$	$\mathbf{N}_{atoms}$	$\mathbf{N}_{sampled}$	$\mathbf{T}_{training}$ **(K)**	$(\mathbf{N}_{atoms})_{ss}$
1	[*]CCC=C([*])CCCCCCC	7	225	615	600-780	6075
2	[*]CC([*])OCCCCCCCC	8	225	2369	600-780	6075
3	[*]CC([*])CCCCC	10	218	514	600-780	5886
4	[*]CC([*])CCC	15	233	542	600-780	6291
5	[*]CC[*]	33	206	1000	600-780	5562
6	[*]CC([*])C	25	233	810	600-780	5592
7	[*]CC([*])CC(C)C	14	260	511	600-780	7020
8	[*]CC([*])C1CCCC1	12	236	842	600-780	6372
9	[*]C(c1ccccc1)C[*]	14	232	1782	600-780	6264
10	[*]CC([*])C1CCCCC1	11	250	1315	800-1040	8000
11	[*]CC([*])(C)c1ccccc1	10	236	795	800-1040	7552
12	[*]CC([*])c1ccccc1C(=O)NC	10	232	1676	800-1040	4640

Table S3: OTF-MLFF estimated coefficient of thermal expansion (CTE) values below and above glass transition.

**SN**	**Polymer smiles**	$CTE_{BelowT_g}(\times 10^{-6}K^{-1})$	$CTE_{AboveT_g}(\times 10^{-6}K^{-1})$
1	[*]CCC=C([*])CCCCCCC	19	216.6
2	[*]CC([*])OCCCCCCCC	19.3	106.9
3	[*]CC([*])CCCCC	108.7	240.6
4	[*]CC([*])CCC	71	301.7
5	[*]CC[*]	80.6	523.7
6	[*]CC([*])C	50.3	233.7
7	[*]CC([*])CC(C)C	100.1	193.6
8	[*]CC([*])C1CCCC1	31.4	151.4
9	[*]C(c1ccccc1)C[*]	50	166.5
10	[*]CC([*])C1CCCCC1	37.3	194.9
11	[*]CC([*])(C)c1ccccc1	16.7	84
12	[*]CC([*])c1ccccc1C(=O)NC	14.7	34.1

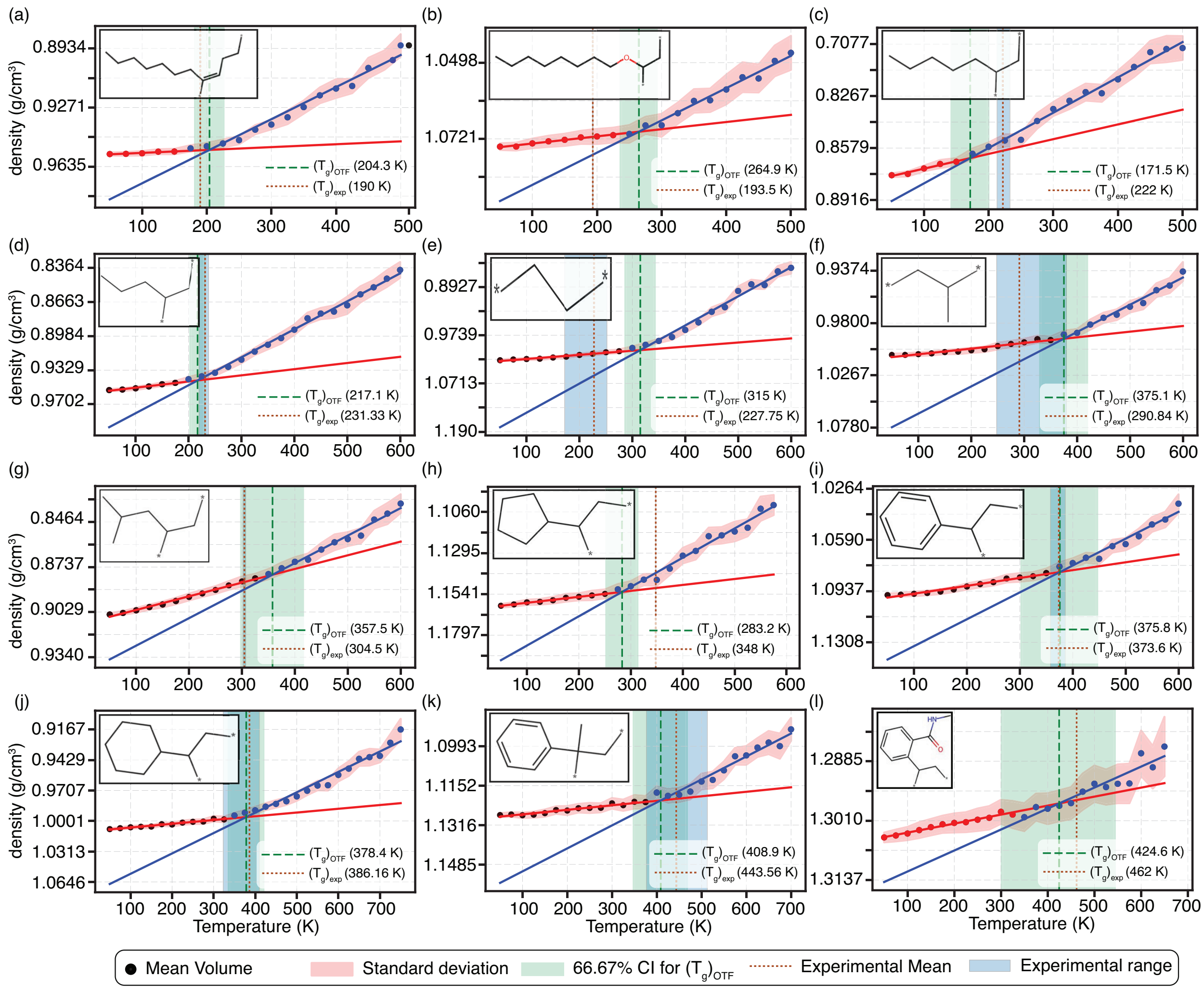

Figure S3: Density-temperature curve of polymers considered in this work. The respective polymer structures have been shown in each plot. Dots (red and blue) and shaded red regions represent the average volumes and standard deviations of the final 50 ps production run of each polymer. Dashed green lines and shaded green regions represent the calculated T_g values and 66.67% confidence interval for T_g (calculated using the error propagation method), respectively. The experimental T_g values have been shown by black dotted lines.
